# Expression of uc.189 and its clinicopathologic significance in gynecological cancers

**DOI:** 10.18632/oncotarget.23761

**Published:** 2017-12-29

**Authors:** Lei Wang, Xing Cheng Wang, Xinyu Li, Yan Gu, Jun Zhou, Shuwan Jiang, Jiajia Liu, Chong Wu, Zhiyan Ding, Yafeng Wan, Chenghai Wang

**Affiliations:** ^1^ Department of Pathology, The Affiliated Hospital of Yangzhou University, Yangzhou University, Yangzhou, Jiangsu 225009, China; ^2^ Department of Pharmacy, Medical College, Yangzhou University, Yangzhou, Jiangsu 225001, China; ^3^ Department of Basic Medical, Medical College, Yangzhou University, Yangzhou, Jiangsu 225001, China; ^4^ Institute of Translational Medicine, Medical College, Yangzhou University, Yangzhou, Jiangsu 225001, China; ^5^ Jiangsu Key Laboratory of Integrated Traditional Chinese and Western Medicine for Prevention and Treatment of Senile Diseases, Yangzhou, Jiangsu 225001, China; ^6^ Jiangsu Co-Innovation Center for Prevention and Control of Important Animal Infectious Diseases and Zoonoses, Yangzhou, Jiangsu 225009, China

**Keywords:** gynecological cancers, cervical squamous cell carcinoma, endometrial adenocarcinoma, uc.189, prognosis

## Abstract

In recent decades, emerging evidence demonstrates that ultraconserved elements (UCEs) encoding noncoding RNAs serve as regulators of gene expression. Until now, the role of uc.189 in human cancers remains undefined and the clinical significance of uc.189 in gynecological cancers remains unknown. This study was to identify the prognostic value of uc.189 expression in gynecological cancers. Tissue microarrays were constructed with 243 samples including 116 cervical squamous cell carcinomas (CSCCs), 98 endometrial adenocarcinomas (EACs), 29 ovarian cystoadenocarcinomas(OCAs), and corresponding normal tissues. In CSCC, uc.189 expression was increased in 78.5% of cases (91/116), decreased in 4.3% (5/116), and unchanged in 17.2% (20/116). In EAC its expression was increased in 74.5% (73/98), decreased in 3.1% (3/98), and unchanged in 22.4% (22/98). Expression of uc.189 was increased in 23, and unchanged/decreased in 6, of 29 cases of ovarian cystoadenocarcinomas. Univariate and multivariate Cox regression analysis demonstrated that over-expression of uc.189 predicted poor prognosis in CSCC and EAC. Thus, these findings suggested uc.189 might be an evaluating prognosis marker of gynecological tumors.

## INTRODUCTION

The incidence of female cancer is increasing, which is a serious threat to women’s health. Among them, cervical cancer, endometrial cancer, ovarian cancer and breast cancer, are the most common malignant tumors. With the development of medical technology, gynecological cancers (GCs) have been studied more and better. For example, cervical cancer and endometrial cancer can be detected early, but it does not reduce its incidence. Ovarian cancer is difficult to detect early, is the leading cause of gynecological cancer deaths. Early detection of molecular markers is the most effective method of GCs prevention; however the molecular mechanisms underlying GCs progression are not well characterized. Therefore, a better understanding of the molecular mechanisms involved in GCs development and progression will provide diagnostic and prognostic markers and potential targets for the therapeutic intervention of GCs.

Ultraconserved elements (UCEs) are noncoding gene sequences with strict conserved across among humans, mice and rats. Emerging evidence demonstrates that UCEs can encode non–protein-coding RNAs (ncRNAs) serve as modulators of gene expression [1, 2]. Previous studies showed that transcribed ultraconserved elements (TUCEs) serve as oncogenes or tumor suppressor genes in development of tumor. For example, uc.73 and uc.338 modulates cell proliferation, apoptosis and metastasis in colorectal cancer (CC) cell lines [3, 4]. Uc.206 acts as a novel oncogene by targeting the P53 gene and promoting CC cell growth [5].

Uc. 189 located in 6p21 is a member of the UCEs. Previous studies showed uc.189 were overexpressed in liver cancer and cervical cancer [6, 7]. It indicates that uc.189 might be an oncogenic gene. However, uc.189 has not been investigated in gynecological cancers before. According to that, we hypothesis that uc.189 may be overexpressed in gynecological cancers.

In the current study, the expression of uc.189 in human tumor samples of different GCs including CSCC, EAC, OCA and corresponding normal tissues was identified using UCEs locked nucleic acid (LNA) *in situ* hybridization, and evaluated its relationship with clinicopathologic parameters and prognosis. To the best of our knowledge, this is the first study to investigate the expression of uc.189 and its clinicopathologic significance in gynecological cancers.

## RESULTS

### Expression of uc.189 in CSCC, EAC and OCA

**Figure 1:**
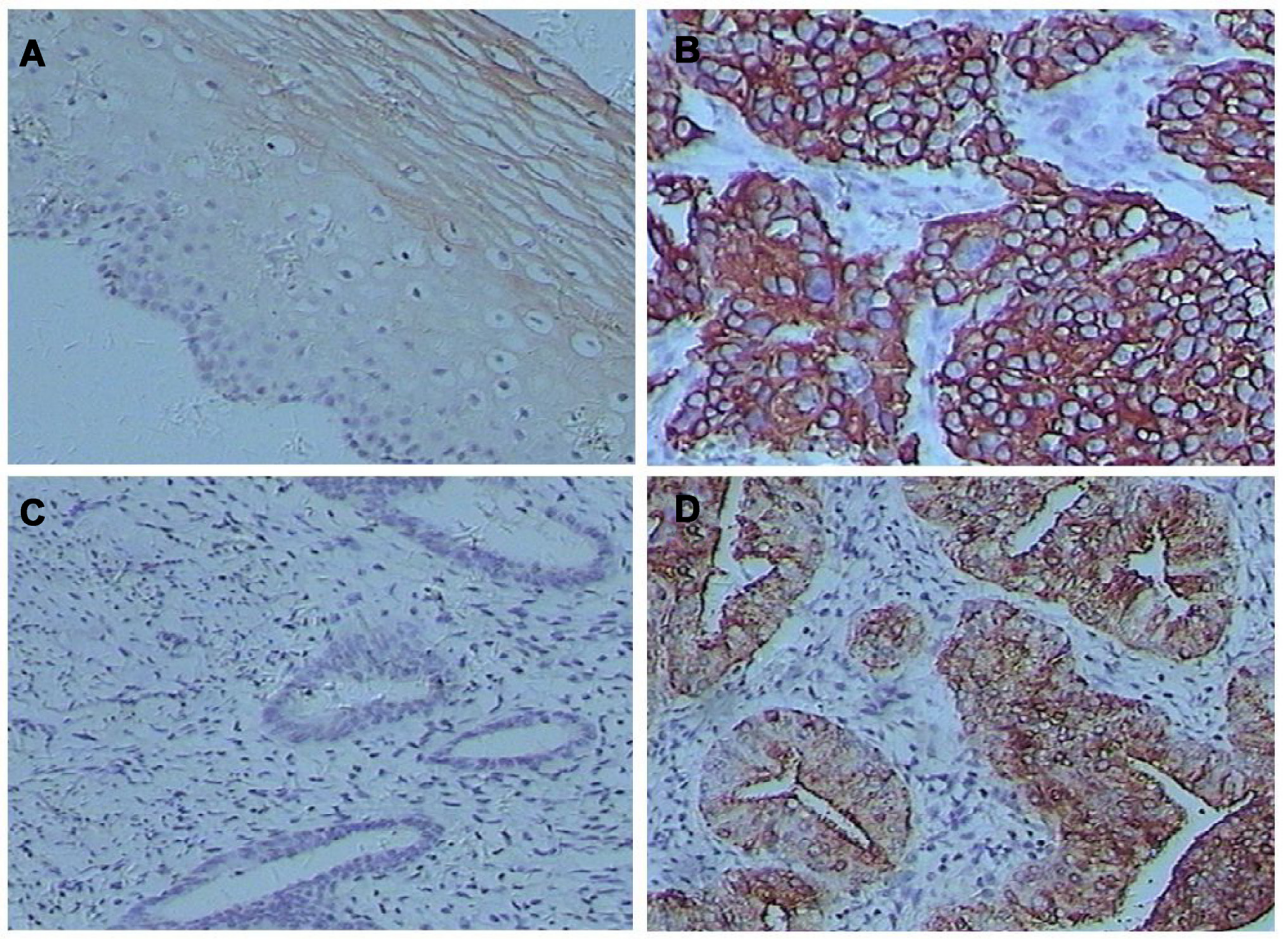
Uc.189 levels were stained by *in situ* hybridization in cervical squamous cell carcinomas (CSCC) and endometrial adenocarcinomas (EAC) (**A**) uc.189 expression in CSCC. (**B**) uc.189 expression in CSCC neighboring normal tissue. (**C**) uc.189 expression in EAC. (**D**) uc.189 expression in EMDC neighboring normal tissue.

The clinic pathologic parameters were listed in Tables 1, 2 and Supplementary Table 1. Uc.189 was mainly located in the cytoplasm of tumor cells from gynecological cancers and neighboring normal tissue (Figure 1). In CSCC, uc.189 expression was increased in 78.5% of cases (91/116), decreased in 4.3% (5/116), and unchanged in 17.2% (20/116). In EAC its expression was increased in 74.5% (73/98), decreased in 3.1% (3/98), and unchanged in 22.4% (22/98). Expression of uc.189 was increased in 23, and unchanged/decreased in 6, of 29 cases of OCA. Aberrant uc.189 expression was thus detected in most gynecological cancer tissues, and its expression was increased in the majority of cases of CSCC, EAC, and OCA (*P* < 0.01).

**Table 1:** Characteristics of the study subjects with cervical squamous cell carcinoma (CSCC)

Clinicopathologic features	Number	Percentage (%)
*Age (years)*		
<60	54	46.6
≥60	62	53.4
*Tumor size (cm)*		
<5	59	50.9
≥5	57	49.1
*Tumor site*		
top half	65	56
bottom half	51	44
*Pathological type*		
Squamous cell carcinoma	62	53.4
Squamous cell carcinoma with CIN	54	46.6
*Pathological grade*		
I	27	23.3
II	51	44
III	38	32.7
*Tumor invasive depth*		
T1 + T2	56	48.3
T3 + T4	60	51.7
*lymph node metastasis*		
negative	41	35.3
positive	75	64.7
*Distant metastasis*		
M0	53	45.7
M1	63	54.3
*Tumor stage*		
I	37	31.9
II	11	9.5
III	36	31.0
IV	32	27.6
*Follow-up time (months)*	6–84	
*Prognosis*		
alive	32	27.6
dead	76	65.5
Unknown	8	6.9
patients lived for ≥ 5 years	65	56
patients lived for < 5 years	44	37.9
Unknown	7	6.1

**Table 2:** Characteristics of the study subjects with endometrial adenocarcinoma (EAC)

Clinicopathologic features	Number	Percentage (%)
*Age (years)*		
<60	33	33.7
≥60	65	66.3
*Tumor size (cm)*		
<5	28	28.6
≥5	70	71.4
*Tumor site*		
Upside	55	56.1
anterior and posterior	43	43.9
*Pathological type*		
adenocarcinoma	54	55.1
adenocarcinoma with dysplasia	44	44.9
*Pathological grade*		
I	19	19.4
II	9	9.2
III	70	71.4
*Tumor invasive depth*		
T1 + T2	52	53.1
T3 + T4	46	46.9
*lymph node metastasis*		
negative	36	36.7
positive	62	63.3
*Distant metastasis*		
M0	41	41.8
M1	57	58.2
*Tumor stage*		
I	11	11.2
II	26	26.5
III	31	31.6
IV	30	30.7
*Follow-up time (months)*	12–84.5	
Prognosis		
alive	32	32.7
dead	58	59.2
unknown	8	8.1
patients lived for ≥ 5 years	52	53.1
patients lived for < 5 years	42	42.9
Unknown	4	4.0

### Relevance of uc.189 expression and clinicopathologic variables in CSCC and EAC

There was a significant difference in Tumor grade (χ^2^ = 10.736, *P* = 0.001), nodal status (χ^2^ = 3.868, *P* = 0.049), TNM stage (χ^2^ = 12.318, *P* = 0.000) between patients with low/ unchanged versus high expression levels of uc.189 in CSCC. In EAC, unchanged/low versus high expression levels of uc.189 also showed a significant difference in tumor size (χ^2^ = 29.518, *P* = 0.000), tumor grade (χ^2^ = 36.993, *P* = 0.000), TNM stage (χ^2^ = 39.674, *P* = 0.002) and distant metastasis (χ^2^ = 40.462, *P* = 0.000). But no significant correlations between uc.189 expression levels and other clinicopathologic variables, including age and depth of tumor invasion (all *P* > 0.05; Tables 3, 4).

**Table 3:** Uc.189 expression and clinicopathologic features in patients with cervical squamous cell carcinoma

Characteristics	Uc.189 low or unchanged (%)	Uc.189 high (%)	χ2	*P*-value
**Age (years)**			0.027	0.870
**<60**	12 (48.0)	42 (46.2)		
**≥60**	13 (52.0)	49 (53.8)		
**Tumor size (cm)**			3.745	0.053
**≥5**	8 (32.0)	49 (53.8)		
**<5**	17 68.0)	42 (46.2)		
**Tumor grade**			10.736	0.001
**I + II**	10 (40.0)	68 (74.7)		
**III**	15 (60.0)	23 (25.3)		
**Local invasion**			1.754	0.185
**T1 + T2**	15 (60.0)	41 (45.1)		
**T3 + T4**	10(40.0)	50 (54.9)		
**Nodal status**			3.868	0.049
**positive**	13 (52.0)	62 (68.1)		
**negative**	12 (48.0)	29 (31.9)		
**TNM stage**			12.318	<0.001
**I + II**	18 (72.0)	30 (33.0)		
**III + IV**	7 (28.0)	61 (67.0)		

**Table 4:** Uc.189 expression and clinicopathologic features in patients with endometrial adenocarcinoma

Characteristics	Uc.189 Low/unchanged expression (*n* = 25)	Uc.189 High expression (*n* = 73)	χ2	*P*-value
**Age (years)**			0.710	0.400
**<60**	3 (21.5)	30 (24.2)		
**≥60**	8 (78.5)	44 (75.8)		
**Local invasion**			1.612	0.204
**T1+T2**	16 (38.1)	36 (19.6)		
**T3+T4**	9 (61.9)	37 (80.4)		
**Tumor grade**			36.993	<0.001
**I + II**	19 (72.4)	9 (5.7)		
**III**	6 (27.6)	64 (94.3)		
**TNM stage**			39.674	<0.001
**I + II**	18 (43.2)	19 (14.8)		
**III + IV**	7 (56.8)	54 (85.2)		
**Nodal status**			0.762	0.383
**positive**	14 (38.9)	48 (16.7)		
**negative**	11 (61.1)	25 (83.3)		
**Distant metastasis**			40.462	<0.001
**M0**	24 (46.3)	17(9.1)		
**M1**	1 (53.7)	56 (90.9)		
**Tumor size (cm)**			29.518	<0.001
**≥5**	7 (64.3)	60 (10.0)		
**<5**	18 (35.7)	10 (90.0)		
**Depth of tumor invasion**			1.612	0.204
**Mucosa, submucosa**	16 (38.1)	36 (19.6)		
**muscularis propria, serosa, adjacent structures**	9 (61.9)	37 (80.4)		

### Survival analysis

In survival analysis, patient follow-up times were ranging from 6–84 months for CSCC and 12–84.5 months for EAC (Tables 1, 2 and Supplementary Table 1). Kaplan–Meier analysis demonstrated that high expression of uc.189, node status, and distant metastasis were significant positive prognostic predictors for OS in patients with CSCC (*P* = 0.000, *P* = 0.001, *P* = 0.000, respectively) and those with EAC (*P* = 0.000, *P* = 0.005, *P* = 0.013, respectively). Other clinicopathologic characteristics, including age, tumor status, location, and TNM stage were not significantly associated with prognosis in CSCC or EAC (*P* > 0.05; Tables 5, 6), but in EAC tumor size and grade were significant difference (*P* = 0.000, *P* = 0.000), there was a tendency towards a difference in TNM stage between patients with high versus low/ unchanged expression levels of uc.189 in CSCC (*P* = 0.083).

**Table 5:** Univariate analysis of survival in cervical squamous cell carcinomas (CSCC)

Variable	Median survival time Month (±SE)	95% CI (Month)	*P*
**Age (years)**			0.230
**<60**	53.18 (2.63)	48.02–58.33	
**≥60**	45.79 (2.99)	39.94–51.64	
**Grade**			0.254
**I–II**	52.80 (2.28)	48.32–57.27	
**III**	43.47 (3.74)	36.14–50.81	
**Stage**			0.083
**I–II**	52.04 (3.45)	45.27–58.81	
**III –IV**	47.86 (2.35)	43.25–52.48	
**Tumor status**			0.440
**T1–T2**	52.90 (2.62)	47.77–58.03	
**T3–T4**	46.73 (2.96)	40.93–52.53	
**Node status**			0.001
**Negative**	58.67 (3.05)	52.70–64.65	
**Positive**	44.69 (2.42)	39.95–49.43	
**uc.189**			<0.001
**High expression**	43.45 (2.04)	39.45–47.44	
**Low/unchanged**	72.47 (1.99)	68.57–76.36	
**Distant metastasis**			<0.001
**M0**	54.61 (3.15)	48.43–60.79	
**M1**	45.34 (2.40)	40.63–50.05	

**Table 6:** Univariate analysis of survival in endometrial cancer (EAC)

Variable	Median survival time Month (±SE)	95% CI (Month)	*P*
**Age (years)**			0.997
**<60**	52.03 (2.66)	46.82–57.24	
**≥60**	49.40 (3.89)	41.79–57.02	
**Tumor size**			<0.001
**≥5**	67.61 (2.11)	63.47–71.75	
**<5**	44.61 (2.56)	39.60–49.62	
**Grade**			<0.001
**I–II**	63.58 (3.78)	56.17–70.99	
**III**	46.08 (2.42)	41.35–50.82	
**Tumor status**			0.898
**T1–T2**	53.84 (2.69)	48.58–59.10	
**T3–T4**	48.10 (3.51)	41.22–54.99	
**Node status**			0.005
**Negative**	60.42 (2.92)	54.70–66.15	
**Positive**	45.70 (2.80)	40.22–51.19	
**Distant metastasis**			0.013
**No**	55.17 (3.7)	47.92–62.42	
**Yes**	48.15 (2.60)	43.06–53.24	
**uc.189**			<0.001
**High expression**	43.52 (2.26)	39.10–47.94	
**Low/unchanged**	72.99 (1.84)	69.39–76.59	
**Stage**			0.374
**I–II**	52.96 (3.88)	45.36–60.56	
**III–IV**	49.97 (2.62)	44.85–55.10	

**Figure 2:**
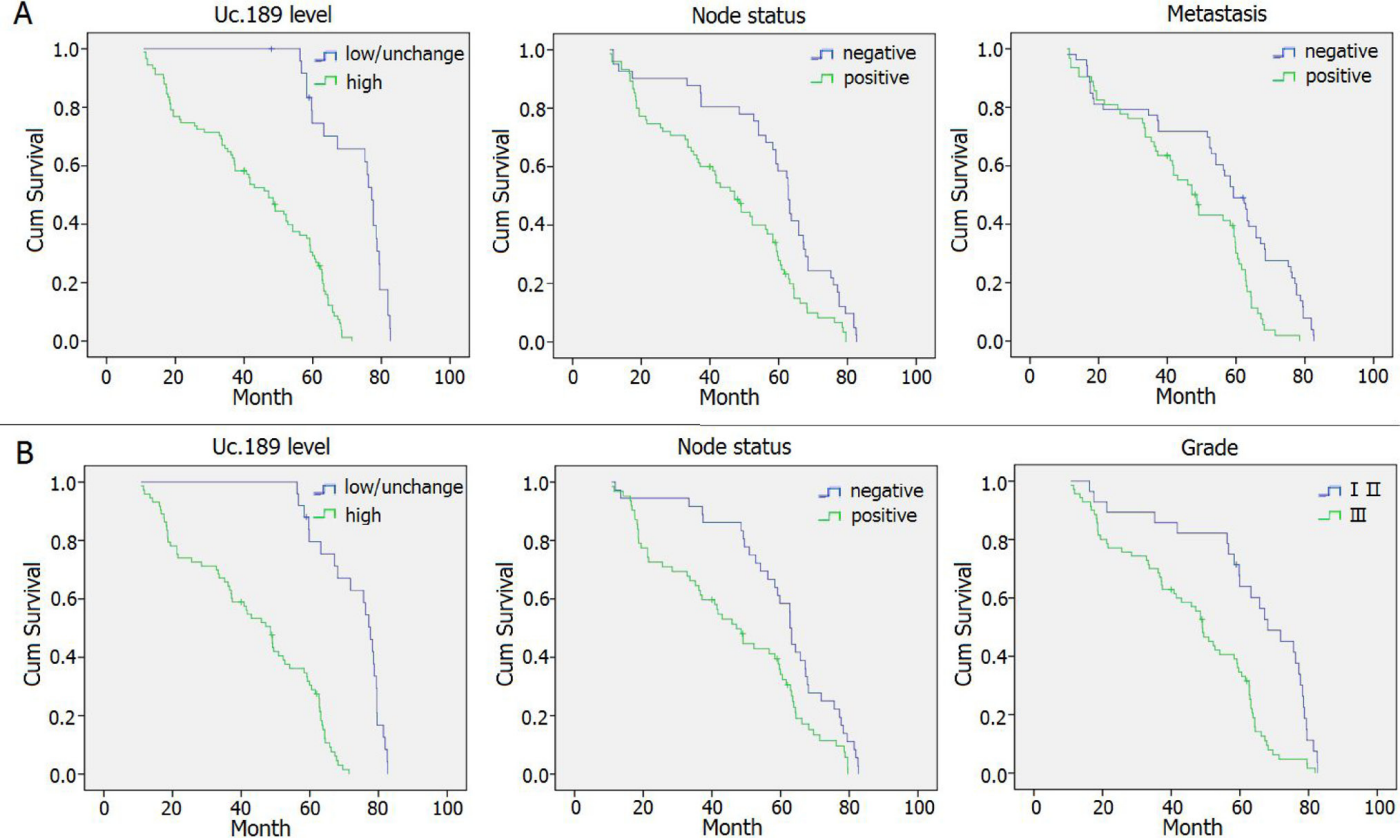
Survival curves in patients with CSCC and EAC according to uc.189 levels (**A**) Overall survival curves in patients with CSCC according to uc.189 levels, node status and metastasis (*P* < 0.05). (**B**) Overall survival curves in patients with EAC according to uc.189 levels, node status and tumor grade (*P* < 0.05).

The prognosis of CSCC patients with high expression of uc.189 was significantly poorer than that of CSCC patients with low/ unchanged uc.189 expression (*P* < 0.001; Figure 2). The median survival times were 43.45 months for high uc.189 expression and 72.47 months for low/unchanged uc.189 expression. Similarly, tumor node status (44.69 vs. 58.67 months; *P* < 0.01, *n* = 116) and distant metastasis (45.34 vs.54.61 months; *P* < 0.01, *n* = 116) also remained a significant predictor of poor survival from Table 5.

The prognosis of EAC patients with high uc.189 expression was dramatically poorer than that of EAC patients with low/unchanged uc.189 expression (*P* < 0.01; Figure 2). The median survival times were 43.52 months for high uc.189 expression and 72.99 months for low/unchanged uc.189 expression. Similarly, tumor node status (44.69 vs. 58.67 months; *P* < 0.01, *n* = 98) and distant metastasis (45.34 vs.54.61 months; *P* < 0.01, *n* = 98) also remained a significant predictor of poor survival from Table 5.

In multivariate analysis for CSCC, tumor stage (HR, 5.825; 95% CI, 1.893–17.930; *P* = 0.002), grade (HR, 0.515; 95% CI, 0.271–0.978; *P* = 0.042), distant metastasis (HR, 0.298; 95% CI, 0.099–0.899; *P* = 0.032), and high uc.189 (HR, 0.067; 95% CI, 0.027–0.165; *P* = 0.000) predicted poor prognosis (Table 7). Moreover, in multivariate analysis for EAC, tumor status (HR,1.759; 95% CI, 1.084–2.854; *P* = 0.022), stage (HR, 2.515; 95% CI, 1.002–6.318; *P* = 0.050), high uc.189 (HR, 0.015; 95% CI, 0.005–0.046; *P* = 0.000), and tumor grade (HR, 2.791; 95% CI, 1.403–5.549; *P* = 0.03) predicted poor prognosis (Table 8).

**Table 7:** Multivariate cox regression analysis of potential prognostic factors for survival in patients with CSCC

Variables	Multivariate analysis	
	HR (95%CI)	*P*-value
**Tumor status, T3-T4 vs. T1-T2**	3.35 (0.937–6.231)	0.580
**Stage, III–IV vs. I–II**	5.825 (1.893–17.930)	0.002
**LNM, yes vs. no**	1.829 (0.866–7.679)	0.267
**Low uc.189 vs. High uc.189**	0.067 (0.027- 0.165)	<0.001
**Tumor size (cm), ≥5 vs. <5**	1.582 (1.120–4.639)	0.29
**Age (years), ≥60 vs. <60**	4.35 (1.237–5.236)	0.488
**Pathological grade, III vs. I II**	0.515 (0.271–0.978)	0.042
**Metastasis, M0 vs. M1**	0.298 (0.099–0.899)	0.032

**Table 8:** Multivariate cox regression analysis of potential prognostic factors for survival in patients with EAC

Variables	Multivariate analysis	
	HR (95%CI)	*P*-value
**Tumor status, T3–T4 vs. T1–T2**	1.759 (1.084–2.854)	0.022
**Stage, III–IV vs. I–II**	2.515 (1.002–6.318)	0.050
**LNM, yes vs. no**	3.613(1.720–8.679)	0.876
**Grade, III vs. I–II**	2.791 (1.403–5.549)	0.003
**Low uc.189 vs. High uc.189**	0.015 (0.005–0.046)	<0.001
**Tumor size (cm), ≥10 vs. <10**	1.904(0.787–4.609)	0.132
**Age (years), ≥60 vs. <60**	1.627(0.720–3.679)	0.257

Abbreviations: *HR* hazard ratio, *CI* confidence interval, *LNM* lymph node metastasis ^*^P < 0.05, statistically significant.

## DISCUSSION

The main common malignant neoplasms of female reproductive system are endometrial carcinoma, cervical and ovarian cancer. Endometrial carcinoma is generally found early, but the prognosis of patients’ with recurrence and metastasis in this cancer is significantly poor [8]. With the wide application of cervical cytological screening, prevention of cervical cancer has made great progress, but chemotherapy is still the main treatment for patients with advanced cervical cancer [9]. The main type of ovarian cancer includes serous, mucinous and endometrial, the survival rate of patients is still low although surgery and drug treatment progress for ovarian carcinomas [10].

Ultraconserved elements (UCEs) are noncoding DNA regions with strict conserved across among mice, rats, and humans. Transcribed UCEs can encode non–protein-coding RNAs (ncRNAs) serve as modulators of transcription and translation of mRNAs [11–13]. By regulating protein production, many UCEs serve as oncogenes or tumor suppressor genes [14, 15]. uc.73 modulates cell proliferation and apoptosis in colorectal cancer cell lines [3]. In bladder cancer tissues, upregulation of uc.8 was inversely related to grade and stage [16], but silence of uc.8 inhibited the proliferation, invasion and migration of cancer cells [17]. Uc.338 is an important oncogene which increased expression of MMP9 to improve invasion and migration of cancer cells in colorectal carcinoma [4], low- expression of uc.73 closely related with stage and grade of colorectal neoplasia [3]. Over-expression of uc.63 suppressed apoptosis of B lymphoma cells and might be predict poor prognosis [18]. Uc.206 acts as a novel oncogene by targeting the P53 gene and promoting CC cell growth, which might be beneficial for cervical cancer therapy [5]. However, little is known about the expression level and biological role of uc.189 in gynecological carcinomas.

This study is the first to systematically explore the expression of uc.189 and its association with clinicopathologic features and prognosis of CSCC, EAC, and ovarian cystoadenocarcinomas, with a view to identifying curative therapies. Our research team found that uc.189 expression was increased in 78.5% of cases (91/116) in CSCC, in EAC its expression was increased in 74.5% (73/98) and over-expression of uc.189 in 79.3% (23/29) of ovarian cystoadenocarcinomas. But in some cases of three types of female carcinomas, uc.189 expression is low or unchanged; this implies heterogeneous expression of uc.189 in a fraction of gynecological system cancers. Moreover, we showed for the first time that uc.189 expression is decreased in most adjacent normal tissues of cervix, endometrial and ovary.

In the present study, CSCC and EAC patients with higher uc.189 expression showed poorer prognoses than those with lower or unchanged expression. Moreover, Kaplan–Meier analysis demonstrated that high expression of uc.189, node status, and distant metastasis were significant positive prognostic predictors for OS in patients with CSCC and those with EAC. In multivariate Cox regression for CSCC, tumor stage, grade, distant metastasis, and high uc.189 (HR, 0.067; 95% CI, 0.027–0.165; *P* = 0.000) predicted poor prognosis. Meanwhile, in multivariate analysis for EAC, stage, high uc.189 (HR, 0.015; 95% CI, 0.005–0.046; *P* = 0.000), and tumor grade predicted poor prognosis.These results showed the detection of uc.189 in tissues of gynecological oncology may provide new information to help in early diagnosis and prognostic evaluation of gynecological cancers, even though the molecular function of uc.189 in those cancers remains unknown. Uc.189 may target to THAP11 according to detection of dual luciferase report system (data unpublished), then cause down-regulation of THAP11. We speculate that down-regulation of THAP11 could promote invasion and migration of cervical cancer Hela cells.

In conclusion, our results indicate that gynecological system cancers, especially cases of CSCC and EAC with high uc.189 expression compared with neighboring normal tissues, have a relatively poor prognosis, whereas low or unchanged uc.189 expression is associated with a better prognosis.

Although further studies are needed to confirm this work, these results suggest that uc.189 may represent a valuable independent prognostic indicator, as well as a potential target for the treatment of gynecological carcinomas.

## MATERIALS AND METHODS

### Tumor tissue collection

A total of 243 samples including 116 cervical squamous cell carcinomas (CSCCs), 98 endometrial adenocarcinomas (EACs), 29 ovarian cystoadenocarcinomas(OCAs), and corresponding normal tissues were used in this study and obtained from 243 patients of gynecological carcinomas (age range, 32–79 years) at the Departments of gynecology of The Affiliated Hospital of Yangzhou University, Yangzhou University (Yangzhou, China). The absence of tumor tissues in the matched normal tissues was confirmed by two pathologists. All tissues were obtained during surgery and immediately stored in liquid nitrogen prior to use. Patients provided written informed consent. The following clinic pathologic data were obtained from the original pathology reports: age, tumor size, location and invasion, lymph node metastases, grade of differentiation, and tumor stage. Staging was assessed according to American Joint Committee on Cancer criteria. Approval for this study was granted by the Institute Research Medical Ethics Committee of The Affiliated Hospital of Yangzhou University, Yangzhou University. Follow-up times were measured from the date of surgery to the date of death for all patients were excluded from uc.189 analysis. The last follow-up point was in December 2016.

### Tissue microarray

Tissue microarrays were constructed using appropriate tissue cores from formalin-fixed and paraffin- embedded samples as described previously [18]. Briefly, appropriate tumor areas and corresponding non-tumor gastric samples were selected by pathologists and a single core (diameter 0.6 mm) was taken from each tissue. Tissue microarray blocks were constructed using an automated tissue arrayer (Beecher Instruments, Sun Prairie, WI, USA). The array blocks were cut into 5-μm sections, and the sections were stained with hematoxylin and eosin to verify the presence of tumor cells. In all cases, tissue cores obtained from normal adjacent tissue served as internal controls.

### *In situ* hybridization

In situ hybridization was performed using Digoxin random primer marker oligonucleotide for uc.189 with scramble- UCE as a negative control and In situ hybridization kit (Boster Biological Technology co. ltd., WuHan, China).A solution of 20 nM probes in hybridization buffer was applied to the array slides, which were covered with Nescofilm (Boster, China) and overnight at 55°C in a humidified chamber. Hybridized probes were detected by incubation with anti-digoxigenin–biotin conjugate at 37°C for 30 min, followed by 3, 3’-diaminobenzidine (DAB) stain to develop a brownish yellow color. Finally, the cells were counterstained with hematoxylin for 3–5 min and mounted on slides.Sections were scored as follows: 0, ≤5% labeled cells; 1, 5–25% labeled cells; 2, 25–75% labeled cells; 3, ≥75% labeled cells. The staining intensity was scored similarly, with 0 indicating negative staining, 1 weakly positive, 2 moderately positive and 3 strongly positive. The scores for the percentage of positive tumor cells and staining intensity were summed to generate an immunoreactive score for each specimen. A final score of 0–1 indicated negative expression (−), 2–3 indicated weak expression (+), 4–5 indicated moderate expression (++), and 6 indicated strong expression (+++). Each sample was examined separately and scored by two pathologists [19–20].

In this analysis, differences in the expression level of uc.189 in tumor tissues are expressed relative to the level of uc.189 in adjacent normal tissues. The expression of uc.189 in normal mucosal samples were judged as positive or negative by the staining status.

### Statistical analysis

All statistical analyses were performed using the SPSS software package (SPSS Inc., Chicago, IL, USA, version 16.0). Associations between clinicopathologic parameters and uc.189 expression were analyzed using χ2-tests. When sample numbers in some categorical cells were lower than 5, the Fisher’s exact test was employed. Overall survival was calculated and survival curves were plotted using the Kaplan–Meier method; differences between groups were compared using log-rank tests. Significant variables in univariate models were further analyzed by multivariate Cox proportional hazards regression models to identify independent prognostic factors. All tests were two- sided and *P* values < 0.05 were considered statistically significant.

## SUPPLEMENTARY MATERIALS TABLE


