# Supplementary material for: Expression of uc.189 and its clinicopathologic significance in gynecological cancers
Source: Oncotarget. 2017 Dec 29;9(7):7453–63. doi: 10.18632/oncotarget.23761 (PMC5800915; doi:10.18632/oncotarget.23761)
Supplement: Supplementary file 1 [file oncotarget-09-7453-s001.pdf]

Expression of uc.189 and its clinicopathologic significance in gynecological cancers

SUPPLEMENTARY MATERIALS

Supplementary Table 1: Characteristics of the study subjects with OCA

Clinicopathologic features	Number	Percentage (%)
Age (years)		
<60	9	31.0
≥60	20	69.0
Tumor size (cm)		
<5	12	41.4
≥5	17	58.6
Pathological type		
serous cystadenoma	7	24.1
serous papillary cystadenocarcinoma	22	75.9
Pathological grade		
I + II	5	17.2
III	24	82.8
Tumor invasive depth		
T1 + T2	11	37.9
T3 + T4	18	62.1
lymph node metastasis		
negative	13	44.8
positive	16	55.2
Distant metastasis		
M0	26	89.7
M1	3	10.3
Tumor stage		
I	13	44.8
II	6	20.7
III	7	24.1
IV	3	10.4
Follow-up time (months)	6–84	
Prognosis		
alive	9	31.0
dead	15	51.7
unknown	5	17.3
patients lived for ≥ 5 years	16	55.2
patients lived for < 5 years	11	37.9
Unknown	2	6.9
